# Resting-State fMRI in Predicting Response to Treatment With SSRIs in First-Episode, Drug-Naive Patients With Major Depressive Disorder

**DOI:** 10.3389/fnins.2022.831278

**Published:** 2022-02-16

**Authors:** Aixia Zhang, Xin Wang, Jianying Li, Lin Jing, Xiaodong Hu, Hejun Li, Chunxia Yang, Kerang Zhang, Ning Sun

**Affiliations:** ^1^Department of Psychiatry, First Hospital of Shanxi Medical University, Taiyuan, China; ^2^First Clinical Medical College of Shanxi Medical University, Taiyuan, China; ^3^Department of Mental Health, Shanxi Medical University, Taiyuan, China

**Keywords:** major depressive disorder, resting-state functional MRI, SSRIs, regional homogeneity, functional connectivity

## Abstract

**Objective:**

For major depressive disorder (MDD), there has been a lack of neuroimaging markers of efficacy of pharmacological treatment. In this study, we aimed to explore the neuroimaging mechanisms in patients with first-episode MDD and identify markers that predict the efficacy of 5-hydroxytryptamine reuptake inhibitors (SSRIs) with the use of resting-state brain imaging technology.

**Methods:**

A total of 101 patients with first-episode MDD and 53 normal controls were finally included in this study. Based on the reduction rate of the score of Hamilton Depression Rating Scale (HAMD-17) during the 2-week SSRI treatment, 31 patients were assigned into the unresponsive group and 32 were assigned into the responsive group. The brain function was compared between patients with MDD and normal controls, and the diagnostic value of brain function was analyzed. With brain regions showing differences between patients with MDD and normal controls as a mask, and the brain function between the responsive and unresponsive groups were compared. Correlations between brain function the HAMD-17 score reduction rate during the 2-week SSRI treatment were analyzed.

**Results:**

Compared to normal controls, patients with MDD showed increased ReHo in the left parahippocampal gyrus and right parahippocampal gyrus, decreased ReHo in the right middle occipital gyrus, and decreased functional connectivity between the right and left parahippocampal gyri, right middle occipital gyrus and middle temporal gyrus. Receiver operator characteristic (ROC) curve analysis showed that the area under the curve (AUC) was 0.544 (95% CI: 0.445–0.644) for ReHo and 0.822 (95% CI: 0.734–0.909) for functional connectivity. Logistic regression pooling of the differences in ReHo mean time series with the functional connectivity mean time series was performed for the ROC curve analysis, which showed an AUC of 0.832 (95% CI: 0.752–0.911). Compared to the responsive group, the unresponsive group showed elevated ReHo in the right parahippocampal gyrus and lower functional connectivity in the middle temporal gyrus. We also found that the ReHo value was negatively correlated with the HAMD-17 score reduction after 2 weeks of SSRI treatment.

**Conclusion:**

Altered resting-state brain function in some regions might be a neurobiological marker for the diagnosis of MDD, and ReHo values are expected to be predictors of patient response to treatment with SSRIs.

**Clinical Trial Registration:**

[http://www.chictr.org.cn/], identifier [ChiCTR1900028722].

## Introduction

Major depressive disorder (MDD) is one of the most common psychiatric disorders associated with severe impairments of quality of life and social function. However, the response and remission rates of MDD remain low as the pathogenesis is still unclear at present. In clinical practice, only 30% of patients are clinically cured by medication, with an response rate of 40–50% (Trivedi et al., 2006; Rush, 2007). Approximately 30% of patients develop refractory depression, and around 70% of patients require several courses of treatment or repeated “trial and error” treatment to achieve remission (McIntyre et al., 2014). A study showed that symptom improvement after 2 weeks of treatment with antidepressants was predictive of clinical remission after 6–8 weeks of treatment, and that patients with MDD who did not respond to antidepressant treatment for 2 weeks had a final remission rate of only 4% if the regimen was unchanged (Szegedi et al., 2009). *Post-hoc* analyses of several clinical trials have also shown that non-response at the end of 2 weeks of antidepressant treatment was predictive of poor treatment outcome after 6–8 weeks (Szegedi et al., 2003; Posternak and Zimmerman, 2005; Papakostas et al., 2006; Taylor et al., 2006; Stassen et al., 2007; Henkel et al., 2009; Hennings et al., 2009; van Calker et al., 2009; Tadić et al., 2010). A study illustrated that non-response at the end of 2 weeks of treatment with 5-hydroxytryptamine reuptake inhibitors (SSRIs) can be considered a valid predictor of poor treatment outcome, indicating that an alternate medication or a combination of drugs could be commenced (Tadić et al., 2016). Therefore, understanding the neuropathological mechanisms of MDD and exploring biomarkers of early therapeutic responses in patients with MDD can provide a basis for clinicians to develop treatment strategies in clinical practice.

Resting-state functional magnetic resonance imaging (MRI) studies have mainly focused on the differences in functional brain activities at rest, while task-state functional MRI studies mainly focused on functional brain activities during tasks; these studies have been proven to be reproducible for the brain imaging procedures and verifiable for the results. At present, the studies of brain function mainly focus on evaluating local functional changes; with brain regions of interest (ROIs) as connection nodes, the spontaneous activities between brain regions are observed, which are used as connection coefficients of brain function to reflect the functional connection between brain regions. Regional homogeneity (ReHo) is used as a measure of regional synchronization of the functional magnetic resonance imaging (fMRI) time course, and has been widely used in clinical studies on MDD. Previous studies on MDD based on resting-state fMRI have revealed abnormalities in several brain regions and functional connections, with more consistent findings involving the prefrontal-amygdala-striatal-medial thalamic regions in the emotion regulation loop. Subsequent studies showed that increased activity in the amygdala and ventrolateral prefrontal cortex at baseline was predictive of poor response to antidepressants, while increased activity in the hippocampus was associated with improvement in depressive symptoms (Williams et al., 2015). During the treatment of MDD with duloxetine, improvement in depressive symptoms was associated with reduced orbitofrontal functional connectivity in the default network; in contrast, reduced orbitofrontal functional connectivity in the default network was associated with poor response to escitalopram in elderly patients with MDD. Meanwhile, increased levels of functional connections in the orbitofrontal cortex prior to treatment were associated with better response to antidepressants (Pizzagalli, 2011). A study showed that a single dose of antidepressants was fast enough to cause significant changes in functional connections in the brain (Schaefer et al., 2014); through brain imaging scans on healthy subjects before and after administration of a single dose of 5-hydroxytryptamine reuptake inhibitors (SSRIs), this study found that functional connections in their brains significantly altered within 3 h. In this study, the whole-brain analysis showed that single doses of 5-hydroxytryptamine reuptake inhibitors (SSRIs) rapidly reduced the level of internal functional connectivity in most brain regions; however, in the cerebellum and thalamus, the level of brain functional connections was increased. It was evident that resting-state fMRI technology has the potential to reflect and identify objective neurobiological markers of psychiatric disorders, and can be used to determine which indicators to use for the early diagnosis and outcome prediction of psychiatric disorders.

## Materials and Methods

### Collection and Evaluation of Clinical Data

#### Subject Selection

A total of 167 first-episode and treatment-naive patients with MDD were recruited from the inpatient and outpatient department of mental health of the First Hospital of Shanxi Medical University from September 2009 to December 2018. All the patients were assessed at baseline with the use of MRI and symptom scales, including the Hamilton Depression Rating Scale (HAMD-17) to assess depressive symptoms and the Hamilton Anxiety Rating Scale (HAMA) to assess anxiety symptoms. Eighty healthy controls were recruited from the community and the university, and none of them were relatives of the patients. All the participants provided informed consent.

Patients with MDD were diagnosed and screened by two experienced psychiatrists with the following inclusion criteria: (1) Han Chinese; (2) aged 18–60 years; (3) right-handed; (4) diagnosed with first-episode MDD based on the DSM-IV criteria and untreated; (5) HAMD-17 score > 17 and HAMA-14 score < 14; (6) having provided informed consent for this study. The exclusion criteria were: (1) with MDD or bipolar disorder secondary to organic diseases or antipsychotic drugs; (2) meeting the DSM-IV-TR criteria for Axis I disorders such as alcohol or drug dependence, traumatic stress, and schizoid affective disorder; (3) with severe organic diseases such as neurological diseases, severe liver and kidney dysfunction, cardiovascular diseases, and craniocerebral trauma; (4) with severe suicidal and self-injurious thoughts, history of suicide attempts (suicide-related score ≥ 2 in the HAMD-17), obvious impulsivity, or uncooperativeness; (5) breastfeeding or pregnant women; (6) with contraindications to the MRI scan.

#### Treatment for the Subjects

The untreated patients with first-episode MDD were given standardized antidepressant medication after enrollment. The drugs were SSRIs, including fluoxetine dispersible tablets (Eli Lilly; 10–40 mg/day), escitalopram tablets (Janssen; 5–20 mg/day), citalopram tablets (Envac; 10–40 mg/day), and sertraline tablets (Pfizer; 25–200 mg/day). All the drugs were initiated at small doses and adjusted based on the patients’ own conditions. Patients with insomnia were given short-term benzodiazepines or supportive psychotherapy as appropriate. Other antidepressants, antipsychotics, electroconvulsive therapy, or other physical therapies were not used within the 2-week treatment period in this study. The patients’ symptoms were evaluated and recorded before treatment and at 2 weeks after the initiation of treatment.

#### Clinical Data Collection

##### General Demographic Information

The demographic information of participants, including sex, age, education, marital status, smoking, alcohol consumption, substance abuse, family history, etc., was recorded using the case report form (CRF) developed by our department.

##### Scales for Clinical Symptoms

The Hamilton Depression Rating Scale (HAMD-17) was used to assess depressive symptoms of the patients and the HAMA was used to assess anxiety symptoms.

### Resting-State Functional Magnetic Resonance Imaging

The MRI scans were performed using a Magnetom Trio (A Tim System) 3T whole-body magnetic resonance imaging device manufactured by Siemens. All the subjects underwent MRI scanning after fully informed of the procedure, approximate time required, and possible adverse reactions to the examination. At the time of scanning, the subjects were placed in a supine position, had their heads fixed with sponge pads and wore headphones to reduce the noise they hear. They were required to keep their eyes closed while remaining awake, and refrain from talking, moving or falling asleep. The subjects were also given an alarm bell to end the scan if they were intolerant. A cranial localization scan was first performed, followed by a resting-state scan and a whole-brain stereo 3D high-resolution T1-weighted scan.

A total of 32 layers were obtained with the use of the following parameters: TR (repetition time) = 2,000 ms, TE (echo time) = 30 ms, FOV (field of view) = 240 × 240 mm2, FA (flip angle) = 90°, acquisition matrix = 64 × 64, THK (thickness) = 3 mm, and gap = 3.99 mm. The subjects were scanned at a total of 212 time points, with the duration of scanning being 8 min and 6 s.

### Analysis of Resting-State Functional Magnetic Resonance Imaging Data

#### Preprocessing of Resting-State Functional Magnetic Resonance Imaging Data

The resting-state fMRI data was preprocessed using DPARSF^1^ based on the SPM8 software (Chao-Gan and Yu-Feng, 2010). The procedures are as follows: (1) the first 10 time points were discarded to allow the magnetic field to reach a steady state; (2) a time-layer correction was performed to exclude the discrepancies caused by intermittent image acquisition; (3) head movement correction was performed by aligning images at all time points to the first image to exclude possible head movements; (4) the single shot echo planar imaging (EPI) template was used for spatial normalization, and the data were normalized to montreal neurological institute (MNI) space and resampled to achieve a voxel size of 3 × 3 × 3 mm^3^; (5) the images were smoothed with a 6-mm FWHM (full-width at half maximum) kernel for subsequent analysis of functional connection; however, for the ReHo (regional homogeneity) analysis, no smoothing was performed; (6) the smoothed data were filtered for frequencies of 0.01–0.08 Hz; (7) the filtered images were delinearized to remove the drift; and (8) regressions were performed on six cephalomotor parameters, as well as cerebrospinal fluid and white matter signals using the preprocessed data. As regression of whole-brain signals would exaggerate the negative correlations between functional connections, it was not performed to ensure the stability of the results. Finally, ReHo and ROI-based functional connection measures were calculated.

The image quality of raw data was checked by experienced neuroimaging physicians to exclude obvious anatomical abnormalities and artifacts in the MRI data of all participants. Then, all images normalized in data space were checked for obvious alignment errors during data preprocessing. Data with artifacts or non-standard alignments were excluded after inspection. Finally, subjects with head movements greater than 2.0 mm in the x, y, and z directions or rotations greater than 2.0 degrees were excluded.

#### Statistical Analyses

After exclusion of patients with unusable data, a total of 101 patients with MDD and 53 normal controls were included in the study. Data analyses were performed using SPM8 software-based DPARSF (see text footnote 1), and independent samples *t*-test was used to compare ReHo, low-frequency oscillatory amplitudes (ALFF)/low-frequency amplitude ratios (fALFF), and brain regions with significant differences in whole-brain functional connections between patients and normal controls. The ROI-based whole-brain functional connectivity was calculated and Fisher’s z transformation was performed using the Gaussian random field (GRF) method. For all the above analyses, *P* < 0.05 indicated statistical significance. Then, receiver operator characteristic (ROC) curves were used to analyze the diagnostic value of resting-state fMRI for MDD.

After 2 weeks of treatment with SSRIs, the patients were divided into the unresponsive group (*n* = 31) and the responsive group (*n* = 32) based on their HAMD-17 scores (unresponsive: reduction rate ≤ 20%, responsive: reduction rate ≥ 50%). With brain regions showing significant difference between patients and normal controls as masks, independent samples *t*-test was performed to compare ReHo, ALFF/fALFF, and ROI-based functional connectivity across the whole brain at baseline between the responsive and unresponsive groups. After the ROI-based functional connectivity was calculated, Fisher’s Z transformation was performed, with AlphaSim used for correction. The threshold for statistical significance for a single voxel was set at *p* < 0.01 (uncorrected) and *p* < 0.05 (corrected). Finally, the mean time series of the brain regions with difference between the responsive and unresponsive groups were extracted and used in a partial correlation analysis with the rate of HAMD reduction during the 2-week treatment, with age, sex, and education as covariates.

## Results

### General Demographic Data and Clinical Characteristics

A total of 101 patients with MDD and 53 normal controls with satisfactory resting-state fMRI data were included in the analyses. The general demographic data and clinical characteristics of the patients with MDD and normal controls are presented in Table 1. According to results of independent samples *t*-test for age and education and chi-square test for sex, there were no significant differences in age (*t* = −0.236, *p* = 0.814), sex (*x*^2^ = 0.104, *p* = 0.747), and education (*t* = 0.980, *p* = 0.329) between the two groups.

**TABLE 1:** ∣ General demographic information and clinical characteristics of the patients with major depressive disorder (MDD) and normal controls.

Variables	MDD patients (*n* = 101)	HCs (*n* = 53)	*P*-value
Age, years ($\bar{\text{x}}$ ± s)	34.50 ± 11.091	35.92 ± 9.23	0.814^a^
Sex (F/M)	56/45	26/27	0.747^b^
Education, years ($\bar{\text{x}}$ ± s)	4.25 ± 1.39	4.47 ± 1.20	0.329^a^
HAMD-17 scores ($\bar{\text{x}}$ ± s)	23.4 ± 2.3	NA	–

*^a^t-test.*

*^b^χ^2^ test.*

*Education: 1 = illiterate, 2 = elementary school, 3 = junior high school, 4 = senior high school, 5 = junior college, 6 = undergraduate, 7 = graduate and above.*

After 2 weeks of SSRI treatment, the patients were divided into the unresponsive group (*n* = 31) and the responsive group (*n* = 32) based on their HAMD-17 scores. The general demographic data and clinical characteristics of the responsive and unresponsive groups are presented in Table 2. According to results of independent sample *t*-test for age, education and HAMD-17 scores and chi-square test for sex, there were no significant differences in age (*t* = −0.566, *p* = 0.577), sex (*X*^2^ = 1.724, *p* = 0.189), and education (*t* = 1.175, *p* = 0.245) between the two groups.

**TABLE 2:** ∣ General demographic data and clinical characteristics of the responsive and unresponsive groups.

Variables	The effective group (*n* = 32)	The ineffective group (*n* = 31)	*P*-value
Age, years ($\bar{\text{x}}$ ± s)	34.59 ± 10.21	36.29 ± 12.49	0.577^a^
Sex (F/M)	18/14	13/16	0.189^b^
Education, years ($\bar{\text{x}}$ ± s)	4.48 ± 1.57	4.10 ± 1.51	0.348^a^
HAMD-17 scores (0 w) ($\bar{\text{x}}$ ± s)	21.74 ± 4.03	20.65 ± 3.05	0.245^a^
HAMD-17 scores (2 w) ($\bar{\text{x}}$ ± s)	7.58 ± 3.75	17.92 ± 3.37	0.000^a^

*^a^t-test.*

*^b^χ^2^ test.*

*Education: 1 = illiterate, 2 = elementary school, 3 = junior high school, 4 = senior high school, 5 = junior college, 6 = undergraduate, 7 = graduate and above.*

### Resting-State Functional Magnetic Resonance Imaging Results

#### ALFF/fALFF

The result of independent samples *t*-test showed that there was no significant difference in ALFF/fALFF between patients with MDD and the normal controls.

#### ReHo

**FIGURE 1:**
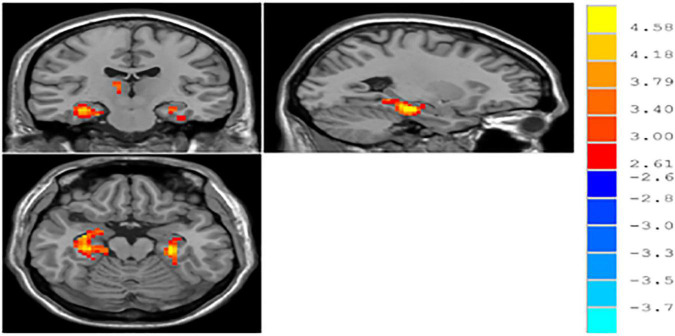
∣ Brain regions with different ReHo between patients with major depressive disorder (MDD) and normal controls.

The result of independent samples *t*-test showed that, after GRF correction, ReHo significantly increased in the left parahippocampal gyrus and right parahippocampal gyrus and significantly decreased in the right middle occipital gyrus in patients with MDD, as compared with normal controls (*p* < 0.05) (see Table 3 and Figure 1).

**TABLE 3:** ∣ ReHo of brain regions showing significant difference between patients with major depressive disorder (MDD) and normal controls.

Regions	Voxel size	BA	Left/ Right	MNI peak coordinates			Peak *t* value
				x	y	z	
**MDD > NCs**							
Parahippocampal gyrus	456	–	Left	−27	−27	−18	4.837
	119	–	Right	9	−24	6	4.572
**MDD < NCs**							
Interoccipital gyrus	176	–	Right	27	−84	24	−4.122

#### Differences in Regions of Interest-Based Functional Connections Between the Patients With Major Depressive Disorder and Normal Controls

**FIGURE 2:**
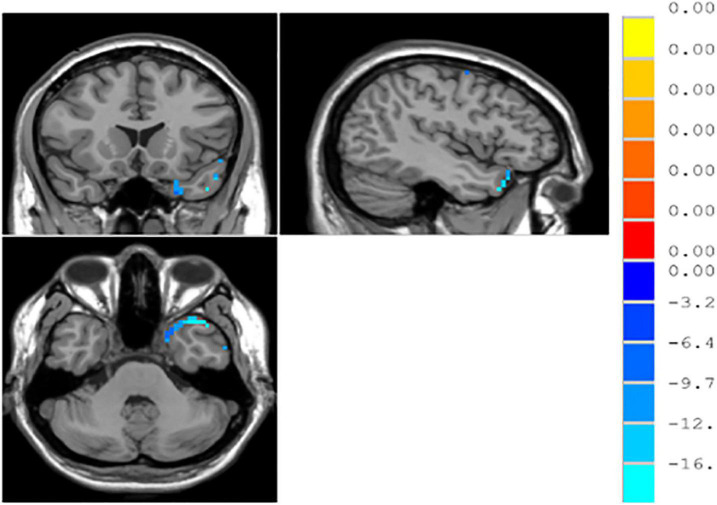
∣ Regions of interest (ROI)-based functional connections in patients with major depressive disorder (MDD) and normal controls.

According to the independent samples *t*-test with GRF correction, the functional connections between the left and right parahippocampal gyri and between the right middle occipital gyrus and middle temporal gyrus significantly decreased in patients with MDD, as compared with normal controls (*p* < 0.05) (see Table 4 and Figure 2).

**TABLE 4:** ∣ Differences in regions of interest (ROI)-based functional connections between patients with major depressive disorder (MDD) and normal controls.

Regions	Voxel size	BA	Left/ Right	MNI peak coordinates			Peak *t* value
				x	y	z	
**MDD < NCs**							
Temporal pole: middle temporal gyrus	57	–	Right	42	21	−39	−23.081
	44	–	Left	−45	18	−36	−19.406
Middle temporal gyrus	56	–	Left	−69	−18	−9	−12.79

**FIGURE 3:**
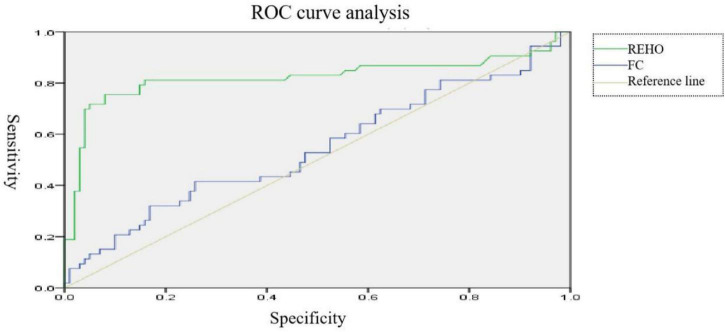
∣ ROC curves for patients with major depressive disorder (MDD).

**FIGURE 4:**
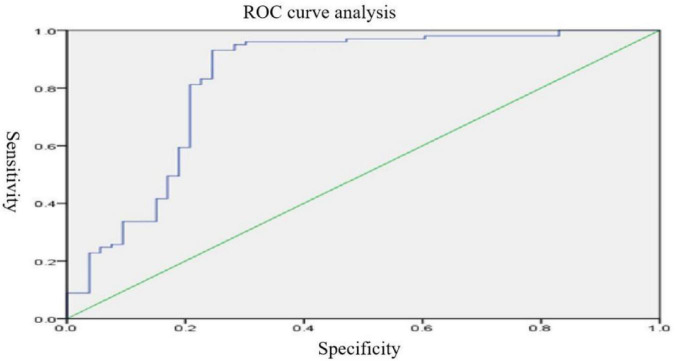
∣ Resting-state ROC curves for major depressive disorder (MDD).

ROC curve analysis was performed by extracting brain regions with different ReHo values with the mean time series of the whole brain functional connections in patients with MDD and normal controls. The curves were plotted with the horizontal coordinate as (1–specificity) and the vertical coordinate as sensitivity. The Youden index of the ROC curve was further calculated as sensitivity + specificity −1. The maximum value of the Youden index was used as the basis for the optimal threshold to calculate the sensitivity and specificity. As shown in Figure 3, the area under the curve (AUC) for ReHo was 0.544 (95% CI: 0.445–0.644), and the AUC for functional connectivity was 0.822 (95% CI: 0.734–0.909). Logistic regression of mean time series of ReHo with the mean time series of functional connections was performed for the ROC curve analysis, which showed that the AUC was 0.832 (95% CI: 0.752–0.911), with a sensitivity of 93.1% and specificity of 75.5% (see Figure 4).

### Comparison of Resting-State Brain Function Between the Responsive and Unresponsive Groups

#### ALFF/fALFF

There were no significant differences in brain areas between the effective and ineffective groups in the ALFF/fALFF ratio using the independent samples *t*-test.

#### ReHo

**FIGURE 5:**
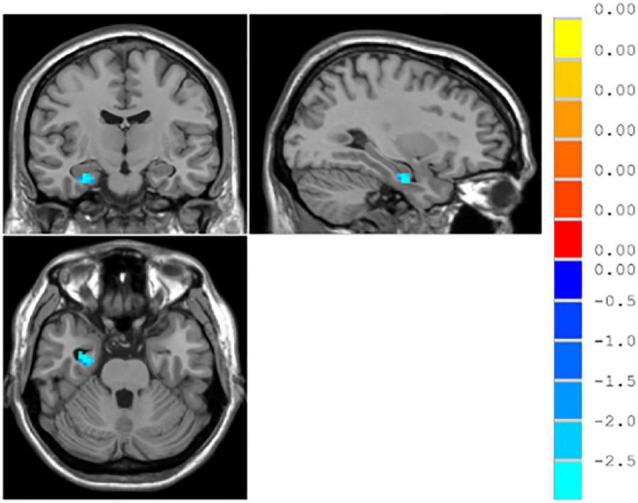
∣ Brain regions with different resting-state ReHo between the responsive and unresponsive groups.

ReHo values in the right parahippocampal gyrus significantly reduced in the responsive group as compared with the unresponsive group, while no significant difference was found in the right middle occipital gyrus and the left parahippocampal gyrus, as shown in Table 5 and Figure 5.

**TABLE 5:** ∣ Brain regions with different resting-state ReHo between the responsive and unresponsive groups.

Regions	Voxel size	Left/ Right	MNI peak coordinates			Peak *t* value
			x	y	z	
**The effective group < ineffective group**						
Parahippocampal gyrus	18	Right	30	−12	−27	−3.1133

#### Regions of Interest-Based Functional Connectivity

**FIGURE 6:**
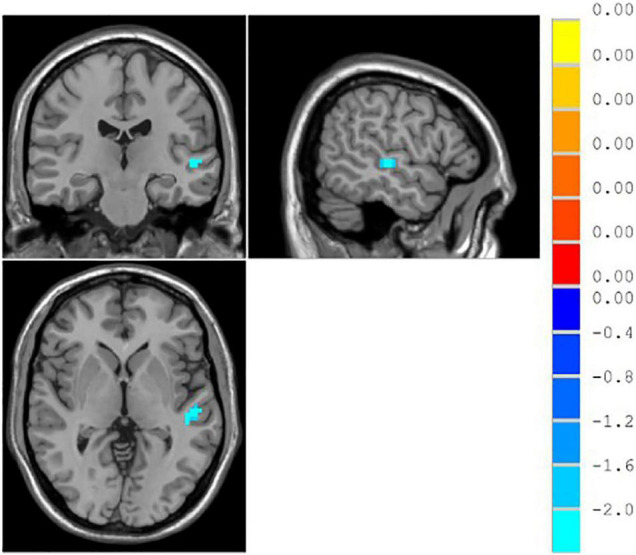
∣ Brain regions with different functional connectivity between the responsive and unresponsive groups.

The result showed that functional connections of the middle temporal gyrus in the unresponsive group were significantly lower than those in the responsive group (*p* < 0.05), as shown in Table 6 and Figure 6.

**TABLE 6:** ∣ Brain regions with different functional connectivity between the responsive and unresponsive groups.

Regions	Voxel size	BA	Left/ Right	MNI peak coordinates			Peak *t* value
				x	y	z	
**The effective group < ineffective group**							
Middle temporal gyrus	21	–	Left	−54	−21	0	−2.4946

### Correlations Between Changes in Resting-State Brain Function and Treatment Efficacy

**FIGURE 7:**
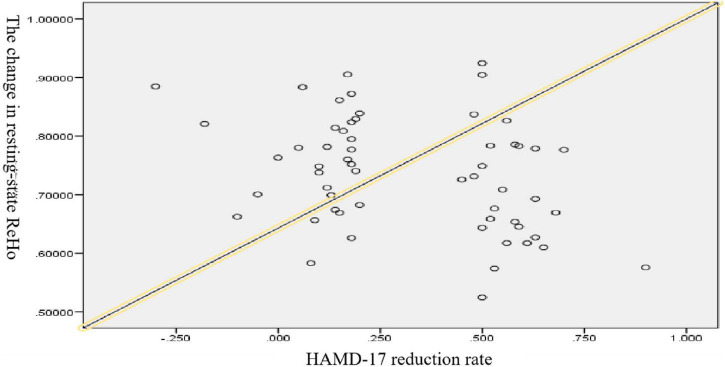
∣ Scatterplot of major depressive disorder (MDD) based on ReHo and HAMD-17 score reduction rate.

The result showed that there was a negative correlation between changes in resting-state ReHo and the HAMD-17 score reduction rate when controlling for sex, age, and education (*r* = −0.265, *p* = 0.028), as shown in Figure 7.

#### Correlation Between Regions of Interest-Based Functional Connectivity and Treatment Efficacy

According to the analysis, no correlation was found between changes in resting-state functional connectivity and the HAMD-17 score reduction rate (*R* = 0.116, *p* = 0.386).

## Discussion

The limbic system is the center of human emotions, behaviors, and memories, and it helps to control stress responses, attention, sexual instincts, etc. The limbic system consists of a complex set of structures, including the cingulate gyrus, parahippocampal gyrus, hippocampal structures, septa, and piriform lobe. As studies advances, the scope of the limbic system has been gradually expanded, and now includes areas that are similar to cortical structures of the limbic lobe, such as the temporal lobe, the posterior part of the frontal orbital gyrus, the anterior part of the insula, and some subcortical structures that are closer in function and connection, such as the septum, amygdala, hypothalamus, superior colliculus, anterior thalamic nucleus, and medial region of the midbrain tegmentum. The hippocampal structures, parahippocampal gyrus and internal olfactory area, dentate gyrus, cingulate gyrus, papillae, and amygdala are interconnected through the Papez loop and connected extensively to other brain structures, such as the neocortex, thalamus, and brainstem. Therefore, researchers inferred that the role of the limbic system is to enable information exchange between the midbrain, mesencephalon, and neocortical structures. The limbic system is involved in mediating instinctive and emotional behaviors through connections with the hypothalamus and the autonomic nervous system, which regulates involuntary body functions. The limbic system is also involved in higher psychoneurological and visceral activities; if damaged, it can lead to mental disorders such as hallucinations, emotional and memory disturbances, abnormal behaviors, unresponsiveness, and impairment of visceral activities.

In this study, no brain regions significantly differed in ALFF/fALFF between patients with MDD and normal controls; increased ReHo was found in the left parahippocampal gyrus and right parahippocampal gyrus and decreased ReHo was found in the right middle occipital gyrus. With brain regions showing different ReHo values between patients with MDD and normal controls as ROIs, it was found that the functional connectivity between the right and left parahippocampal gyri and between the right middle occipital gyrus and middle temporal gyrus were reduced in the patients with MDD. Previous studies have found increased fALFF values in the left supraoccipital gyrus and decreased fALFF values in the left parahippocampal gyrus in patients with MDD, as compared to normal controls (Liu et al., 2013). Fan et al. (2013) found that patients with MDD, compared to normal controls, had increased ALFF values in the right parahippocampal gyrus and decreased ALFF values in the left angular gyrus and left middle occipital gyrus. With regard to ReHo, a study found that patients with MDD showed reduced ReHo values in the right orbitofrontal cortex, cingulate gyrus, ventral anterior cingulate, posterior cingulate, and insula, as well as in the left dorsal anterior cingulate, nucleus accumbens, thalamus, temporal lobe, posterior cerebellum, and bilateral occipital lobes (Yao et al., 2009). Studies on resting-state functional connectivity in MDD selected different ROIs. Using the cingulate gyrus as the ROI, studies found that the anterior subgenual cingulate gyrus had enhanced connectivity to the dorsomedial frontal lobe and left dorsolateral frontal lobe and decreased connectivity to the insula, amygdala, and precuneus in in patients with MDD (Wang et al., 2012; Connolly et al., 2013). Using the amygdala as the ROI, studies have shown that patients with MDD have decreased functional connectivity of the amygdala with the ventral lateral prefrontal lobe, insula, middle temporal/superior gyrus, cerebellum and occipital lobe and enhanced functional connectivity with the bilateral temporal poles; the amygdala also had reduced functional connectivity with the left ventral prefrontal lobe (Tang et al., 2013; Ramasubbu et al., 2014). Using the hippocampus as the ROI, a study found that patients with MDD had enhanced functional connectivity of the hippocampus with the bilateral limbic system, temporal lobe, and inferior/medial prefrontal lobe and reduced functional connectivity with the bilateral prefrontal, occipital, and parietal lobes, as well as the cerebellum (Cao et al., 2012). ReHo-mean time series and the averaged functionally connected time series were used in the logistic regression for the ROC curve analysis with a discrimination of 83%, indicating that resting-state MRI provided a high diagnostic value for MDD.

This study explored the association between the efficacy of SSRIs for MDD and resting-state brain function. It found that after 2 weeks of treatment with SSRIs, there was no difference in ALFF/fALFF between the unresponsive group and the responsive group, while elevated ReHo values were found in the right parahippocampal gyrus. We also found that the unresponsive group had lower functional connectivity in the middle temporal gyrus. Previous studies have shown that the activity of the bilateral frontal middle lobes, parahippocampal gyrus, and cerebellum might be related to patient responses to drugs (Alexopoulos et al., 2012). Differences in the correlations between resting-state functional connectivity and treatment outcomes might be resulted from different grouping method or analyses of data from patients with different severities of MDD.

In summary, the inconsistent results of resting-state fMRI studies on MDD might be attributed to the following factors. First, the different diagnostic criteria for MDD used in these studies led to differences in study samples; second, the status of patients with MDD was different across studies; and third, the different methods of data collection, processing, and analysis might have an impact on the results. Based on resting-state fMRI imaging, the present study found that the mechanisms of MDD and the prediction of responses to SSRIs might involve abnormalities in brain regions associated with affective disorders, especially in the limbic system, including the hippocampus, parahippocampal gyrus, cingulate gyrus, and temporal lobe.

Despite the strengths in our study, some limitations should also be noted. Firstly, there is a lack of longitudinal MRI data due to great loss to follow-up. A follow-up plan has been developed to expand our sample and prepare for further long-term longitudinal follow-up studies. Secondly, it has been demonstrated in previous studies that the duration of illness before treatment in patients with depression is associated with greater volume loss in some brain regions. However, this was not included the data collected for this study. Thirdly, the range of age in this study was broad, and the effects of brain development and aging were not taken into account. Finally, the reproducibility of the study results was relatively low. Hopefully, future studies using multiple imaging methods and multicenter data can further validate the results of this study and provide guidance for the prediction of treatment efficacy and individualized treatment for patients with MDD.

## Conclusion

Through analyses of the fMRI data and treatment response of patients with MDD, this study suggested that altered resting-state function in some brain regions might be a neurobiological marker for the diagnosis of MDD and that the degree of impairment in resting-state ReHo at baseline is expected to be a predictor of the efficacy of SSRIs in patients with MDD.

## Data Availability Statement

The raw data supporting the conclusions of this article will be made available by the authors, without undue reservation.

## Ethics Statement

The studies involving human participants were reviewed and approved by the First Hospital of Shanxi Medical University. The patients/participants provided their written informed consent to participate in this study. Written informed consent was obtained from the individual(s) for the publication of any potentially identifiable images or data included in this article.

## Author Contributions

KZ and NS designed the experiments. AZ, XW, JL, LJ, XH, HL, and CY performed the clinical data collection and assessment. AZ and XW performed the neuroimaging data analysis and wrote the draft. All the authors discussed the results and reviewed the manuscript.

## Conflict of Interest

The authors declare that the research was conducted in the absence of any commercial or financial relationships that could be construed as a potential conflict of interest.

## Publisher’s Note

All claims expressed in this article are solely those of the authors and do not necessarily represent those of their affiliated organizations, or those of the publisher, the editors and the reviewers. Any product that may be evaluated in this article, or claim that may be made by its manufacturer, is not guaranteed or endorsed by the publisher.

## Footnotes

1
http://rfmri.org/dpabi
